# Whole-Genome Sequence of *Aneurinibacillus* sp. *Ricciae_BoGa-3*, Isolated from Riccia fluitans

**DOI:** 10.1128/mra.00081-23

**Published:** 2023-05-04

**Authors:** Marvin Hildebrandt, Isabell E. Bleile, Felix Althoff, Sabine Zachgo, Andrea Bräutigam, Bart Verwaaijen

**Affiliations:** a Bielefeld University, Computational Biology, Faculty of Biology, Bielefeld, Germany; b CeBiTec, Bielefeld University, Bielefeld, Germany; c Osnabrück University, Department of Botany, Osnabrück, Germany; d Department of Genetics, Martin-Luther-University Halle-Wittenberg, Halle, Germany; University of Arizona

## Abstract

Here, we present the Nanopore-only genome sequence of *Aneurinibacillus* sp. *Ricciae_BoGa-3*. It was isolated from Riccia fluitans ecotype BoGa-3 and its source was Botanical Garden Osnabrück (Germany). The complete circular genome is 4,981,254 bp with a GC content of 44.8%.

## ANNOUNCEMENT

Members of the Gram-positive, endospore-forming, and rod-shaped bacterial genus *Aneurinibacillus* (1) occupy diverse habitats like the plant rhizosphere (2), geothermal soil (3), or marine environments (4). Several *Aneurinibacillus* species are known to produce useful metabolites, such as antibiotics (5) and biosurfactants (4), or exhibit plant-growth-promoting traits, such as phosphate solubilization and growth inhibition of plant pathogens (2). Of this genus, so far, only seven type strains have been published according to the Type Strain Genome Server (TYGS) (6, 7). Therefore, there may be a great potential within this genus for further discoveries of species that have useful properties for agricultural or biotechnological applications (8).

We isolated *Aneurinibacillus* sp. *Ricciae_BoGa-3* from a laboratory-grown Riccia fluitans (floating crystalwort) (9) line, ecotype BoGa, named after its original source, the Botanical Garden of Osnabrück University (Germany) (10, 11). The medium used was 1/2 Gamborg B5 medium with 1% glucose. Plants were grown at room temperature with a 16:8 day:night regime, and after colonies had formed around the plants, DNA was isolated from a single colony, with the NucleoSpin microbial DNA minikit (Macherey-Nagel, Düren, Germany). Sequencing was performed with the kit SQK-LSK109 on a GridION device with a R9.4.1 flow cell (Oxford Nanopore, Oxford, UK). Next, we performed base calling at super accuracy (Guppy v5.0.11), assembly (Canu v2.1.1) (12), and polishing with Racon (v1.4.20) (13) in combination with BWA (v0.7.17) (14) and Medaka (v1.4.3). The final contig was circularized and oriented manually. Default settings were used for all tools.

Raw sequencing generated 1.25 million reads, an *N*_50_ of 9.07 kilobases, and 3.22 total gigabases. Assembly showed 511× coverage, a GC content of 44.8%, and a length of 4,981,254 bp. Annotation showed 5,938 total genes and 5,472 coding genes. Genome completeness was determined with BUSCO and included 98.3% complete (C), 96.7% single copy (S), 1.6% duplicated (D), 1.1% fragmented (F), and 0.6% missing (M) orthologues genes (15, 16). Annotation was based on NCBI PGAP (v6.4) annotation of CP116887 on 1 January 2023 (17).

**FIG 1:**
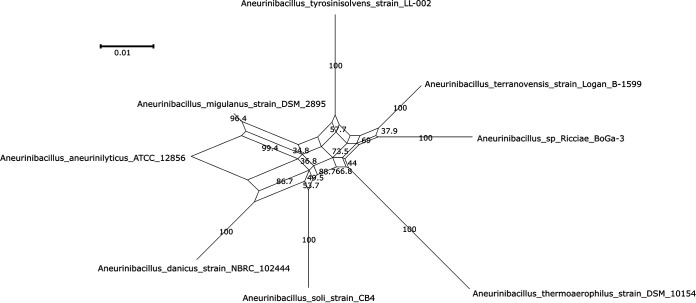
Phylogenetic network of *A. ricciae* and the seven *Aneurinibacillus* type strains calculated with Clustal Omega and SplitsTree (18). The alignment was calculated using 1,500-bp-long 16S RNA sequences. Bootstrap values from 1,000 replications are shown as labels on the corresponding edges.

For the genome sequence of *Aneurinibacillus* sp. *Ricciae_BoGa-3* and the seven published type strains, a phylogenetic network was calculated using SplitsTree (18) (Fig. 1) with default settings on an alignment of 16S sequences created with Clustal Omega (19). Complementary to the 16S-based network, TYGS (6, 7) was used to calculate a whole-proteome-based tree (not shown). The obtained average branch support for the tree is 98.1%. The network and tree both support the finding that *Aneurinibacillus* sp. *Ricciae_BoGa-3* is closest to Aneurinibacillus terranovensis but with enough phylogenetic distance to indicate that *Aneurinibacillus* sp. *Ricciae_BoGa-3* is distinct from previously known species. Neither the addition of not-type-strain species to the network nor a BLAST search against the NCBI nonredundant (nr) database (20, 21) for the 16S sequence provide additional candidates for more closely related species. The digital DNA-DNA hybridization (dDDH) values for *Aneurinibacillus* sp. *Ricciae_BoGa-3* and *A. terranovensis* for all three distance formulas are significantly below the 70% cutoff (22).

Although *Aneurinibacillus* sp. *Ricciae_BoGa-3* formed colonies when cocultivated with its host, it did not grow on 1/2 Gamborg medium with 1% glucose. This finding suggests a dependency on one or more plant exudates. According to a mapping of genes on KEGG pathways (23, 24), the bacterium may be auxotrophic for several B vitamins. These findings combined with the existing literature base of the genus indicate that *Aneurinibacillus* sp. *Ricciae_BoGa-3* is a plant-associated species and possible symbiont. The genome presented here was determined with Nanopore only and despite deep sequencing (511×) might still contain Nanopore sequencing-related errors.

### Data availability.

This whole-genome shotgun project has been deposited in GenBank under the BioProject no. PRJNA914707, BioSample no. SAMN32348813, and accession no. CP116887. Raw sequence reads can be found in SRA under SRR23191365.
